# Oxidant Status following Cardiac Surgery with Phosphorylcholine-Coated Extracorporeal Circulation Systems

**DOI:** 10.1155/2016/3932092

**Published:** 2016-11-23

**Authors:** Ali Can Hatemi, Kadir Çeviker, Aybala Tongut, İlhan Özgöl, Murat Mert, Ayşem Kaya

**Affiliations:** ^1^Department of Cardiovascular Surgery, Institute of Cardiology, Istanbul University, Istanbul, Turkey; ^2^Department of Cardiovascular Surgery, Faculty of Medicine, Süleyman Demirel University, Isparta, Turkey; ^3^Pediatric Cardiac Surgery Clinic, Kartal Kosuyolu YİEAH, Istanbul, Turkey; ^4^Division of Biochemistry, Institute of Cardiology, Istanbul University, Istanbul, Turkey

## Abstract

*Introduction*. Extracorporeal circulation (ECC) related systemic oxidative stress is a well-known entity but the underlying mechanisms are not clearly described. Our aim was to investigate the relation between the oxidative stress indices, inflammatory markers, and phosphorylcholine-coated (PCC) ECC systems.* Patients and Methods*. Thirty-two consecutive coronary artery bypass grafting (CABG) cases were randomly assigned to Group I (PCC, *n* = 18) and Group II (noncoated, *n* = 14) ECC circuits. Total Antioxidant Status (TAS), Total Oxidant Status (TOS), Tumor Necrosis Factor-*α* (TNF-*α*), Interleukin-1*β* (IL-*β*), Interleukin-6 (IL-6), Interleukin-8 (IL-8), Interleukin-10 (IL-10), and Procalcitonin (PCT) levels were measured at 5 different time points. The association between the oxidative indices levels and PCC system used was analyzed.* Results*. In Group I TOS and TAS statuses were increased at *T*1, *T*2, *T*3, and *T*4, while IL-10 and TNF-*α* levels accompanied those raises only at *T*2 (Group I-Group II, 4.73 ± 2.04 versus 2.79 ± 0.63, *p* = 0.002, and 30.56 ± 8.11 versus 23.97 ± 7.8, *p* = 0.031, resp.). In contrast, mean TAS and TOS levels were similar to baseline at all time points in Group II but IL-6 and IL-8 levels were increased at *T*2 (Group I-Group II, 16.84 ± 5.63 versus 44.81 ± 17.0, *p* = 0.001, and 38.88 ± 9.8 versus 46.14 ± 9.25, *p* = 0.038, resp.).* Conclusion*. Even coated ECC systems are still incapable of attenuating the inflammatory response to cardiopulmonary bypass (CPB).

## 1. Introduction

Although cardiac surgery under cardiopulmonary bypass (CPB) has become a routine procedure worldwide, its postoperative complications are still unpredictable. It has been suggested that a significant proportion of the adverse outcomes may also be caused by the systemic effects of CPB [1]. The endothelial injury and/or cardiac, renal, hepatic, or pulmonary dysfunction associated with CPB have been linked to the inflammatory responses and systemic oxidative stress directly caused by ECC system, but the underlying mechanisms have not been fully elucidated yet [2].

Factors responsible for the inflammatory activation can be listed as leucocytes and adhesion molecules, oxygen-free radicals, arachidonic acid products, cytokines, endotoxins, activated thrombocytes, and the complement system. One of the most damaging consequences of these events is the formation of Reactive Oxygen Species (ROS) and radicals, which originate from various cellular and enzymatic sources such as myocardial cells, activated neutrophils, or endothelial xanthine oxidase [3]. Numerous studies describing the nature of oxidant and antioxidant status and the time course of their formation during CPB were published [3, 4]. The antioxidant system which is induced against oxidative stress as a hemostatic mechanism leads in turn to depletion of plasma antioxydants [3]. In order to counterbalance this sequence of events and to diminish oxidative injury, several studies have investigated the use of coating materials such as heparin, PMEA, and phosphorylcholine on the surface of Extracorporeal Circulation Circuit (ECC). Less is known about the effects of the phosphorylcholine-coated (PCC) ECC systems on TAS and TOS.

In order to elucidate the impact of the PCC material on the oxidant and antioxidant status during CPB, we monitored TAS, TOS, and Oxidative Stress Index (OSI) at specific time points. Inflammation markers such as TNF-*α*, IL-*β*, IL-6, IL-8, and IL-10 and procalcitonin levels were also monitored simultaneously.

## 2. Material and Methods

This is a single blinded prospective study in accordance with the principles outlined in the Declaration of Helsinki. Ethical permission was obtained from the local ethical committee and all of the patients had given their written informed consents before the study.

### 2.1. Patient Selection

Thirty-two patients (26 males, 6 females, mean age: 61 ± 10 years) with a diagnosis of ischemic heart disease who underwent elective coronary artery bypass surgery (CABG) with similar demographic characteristics were enrolled in the study group (single blinded) and were randomly divided into two groups on the basis of odd or even number poll preoperatively. Phosphorylcholine-coated open ECC system was used in Group I while noncoated open ECC system was used in Group II.

Exclusion criteria were as follows: low ejection fraction (EF < 30%), emergency operation, LV aneurysm, reoperation, concomitant procedures (e.g., valve replacement, carotid endarterectomy, and left ventricular aneurysm resection), perioperative MI, old age (>70 years), renal insufficiency, and consent denial. Demographic characteristics and postoperative clinical data of patients are shown in Table 1.

### 2.2. Blood Sample Collection

Blood samples for hematologic, biochemical, and immunologic parameters were collected, preoperatively (*T*0), before cross-clamp removal (*T*1), following CPB termination (*T*2), at the 6th postoperative hour (*T*3), and at the 1st postoperative week (*T*4). Clinical signs of inflammations were also followed and recorded until the 7th postoperative day.

Blood samples were kept in gel buffered dry tube and K3 EDTA tube. Complete blood counts were analyzed by automatic 24-parameter blood count device (Bayer Pentra 80-Siemens Healthcare Diagnostics Products, Marburg, Germany). Blood samples in the dry tubes were centrifuged 10 minutes at 5000 rpm, and the plasma obtained was put in a 2 ml cryo-Eppendorf tubes for specific tests. Cryo-Eppendorf tubes were held at −80°C refrigerator until analysis. At the end of the study heating process was applied to all frozen plasma samples and they were analyzed with a Siemens BCS measuring device (Siemens Healthcare Diagnostics Products GmbH 2008 Marburg, Germany).

### 2.3. Anesthetic Protocol

Patients were premedicated with 0.05 mg/kg I.M. midazolam (Dormicum, Roche Pharmaceuticals, Nutley, NJ, USA) 30 minutes before surgery. After premedication, all patients received 6 l/min oxygen via face-mask under noninvasive saturation monitoring. Sixteen-gauge intravenous and intra-arterial (radial artery) catheterizations were performed, following a 1 mg subcutaneous prilocaine (Citanest, Zentiva Sağlık Urünleri San. ve Tic. A.Ş., Kırklareli, Turkey) injection. Continuous 5 leads' ECG monitoring was established. Following induction with 2% lidocaine (Aritmal, Osel Ilaç San. ve Tic. A.Ş., Istanbul, Turkey), midazolam 0.05 mg/kg, fentanyl citrate 25–30 mcg/kg (Abbojet, Abbott Laboratories, Abbott Park, LA, USA), ketamine 1 mg/kg (Ketalar, Zentiva Sağlık Urünleri San. ve Tic. A.Ş., Kırklareli, Turkey), etomidate 0.2 mg/kg (Lipuro, B. Braun Melsungen AG, Berlin, Germany), and pancuronium 0.1 mg/kg (Pavulon, Merck Sharp Dohme Ilaclari Ltd. Şti., Istanbul, Turkey) intubation was performed. Nasoesophageal temperature probe and capnographs were placed, after nasogastric tube and Foley catheter applications. Before ECC commencement, at first 300 IU/kg of systemic heparin was administered and then additional heparin doses were administered to keep Activated Clotting Time (ACT) above 480 seconds (Hemochron 801, ITC, USA) [5]. Anesthesia was maintained with propofol 0.05 mg/kg/min (Diprivan, AstraZeneca Türkiye İlaç Sanayi ve Ticaret Ltd. Şti., Istanbul, Turkey) and remifentanil 25 mcg/kg/min infusion (Ultiva, GlaxoSmithKline Manufacturing S.p.A, Milan, Italy), each 2 hours, with 2 mg of pancuronium administration. No patient in either group required high dose inotrope support after CPB or postoperatively.

### 2.4. Surgical Protocol

All operations were performed by the same surgical team. Following median sternotomy, all patients received left internal thoracic artery and saphenous vein grafts as conduits. Extracorporeal circulation was conducted at 32°C. Proximal anastomoses were created under partial cross-clamp during the rewarming phase. Operations were completed in the standard fashion.

### 2.5. Cardiopulmonary Bypass Technique

Following aorta two-stage cannulation cardiopulmonary bypass was instituted and an antegrade cardioplegia cannula was placed on the ascending aorta. A roller pump (Jostra HL20, Herrlingen, Germany) was used in all cases. All the patients' ECC system consisted of a cardiotomy reservoir, a tubing set, a hollow fiber membrane oxygenator, and a 40 *μ*m arterial line filter. Phosphorylcholine-coated circuits (Compactflo Evo Phisio-Sorin Group Italia, Italy) were used for Group I and conventional noncoated circuits (Bıçakçılar-Bıçakçılar Medical Devices Industry and Trade. Co., Istanbul, Turkey) were used for Group II. Mild hypothermia was applied with a nasopharyngeal temperature of 32°C and a nonpulsatile flow of 2.4 l/min/m^2^ and a mean arterial pressure of 55–65 mmHg was maintained during the ECC. Hemodilution was achieved with a hematocrit level of 26%. The circuits were primed with a mixture of 500 ml succinyl gelatine 6% (Gelofusine IV, B. Braun Medikal Dış Ticaret A.Ş, Esenler, Istanbul, Turkey), 1000 ml isolyte, 100 ml mannitol 20%, NaHCO3 8.4%, 250 mg methylprednisolone (Prednol 250, Mustafa Nevzat Inc., Istanbul, Turkey), and 1 g cefazolin (Cefamezin 1 g, Zentiva Sağlık Urünleri San. ve Tic. A.Ş., Istanbul, Turkey) at room temperature (20°C) adding up to a total of 1600 ml fluid. Myocardial protection was provided via intermittent (20 min intervals) antegrade cold blood cardioplegia. The cardioplegic solution consisted of 2.4 mEq/lt calcium, 32 mEq/l magnesium, 86 mEq/l potassium, 110 mEq/l sodium, 160 mEq/l chlorine, and 10 mEq/l sodium bicarbonate. The cardioplegic solution was instituted at a volume of 1000 ml initially and repeated as 500 ml every 20 minutes, reaching a total of 2000–2500 ml depending on the patient. Anticoagulation was achieved using 300 U/kg of heparin. If required, heparin was supplemented to maintain the ACT above 480 sec. and reversed by protamine hydrochloride at the end of the procedure (Protamin ICN, Mefar A.Ş., Istanbul, Turkey).

### 2.6. Measurement of TAS and TOS and Oxidative Stress Index (OSI)

Serum TAS and TOS levels were determined using a novel automated measurement method, using original kits (Total Antioxidant Status Assay Kit and Total Oxidant status Assay Kit, Rel Assay Diagnostic Clinical Chemistry Solutions, Gaziantep, Turkey) [6, 7]. The antioxidative effect of the study sample against the potent-free radical reactions, which are initiated by the produced hydroxyl radical, is measured. Oxidants, present in the study sample, oxidize the ferrous ionodianisidine complex to ferric ion. The oxidation is enhanced by glycerol molecules, which are abundantly present in the reaction medium. The ferric ion makes a colored complex with xylenol orange in an acidic medium. The color intensity, which can be measured spectrophotometrically, is related to sample's TOS level. Percent ratio of TOS to TAS levels is accepted as Oxidative Stress Index (OSI = TOS (*μ*mol H_2_O_2_ Equiv./l)/TAS (mmol Trolox Equiv./l)).

### 2.7. Procalcitonin

Procalcitonin levels were determined using Vidas Brahms Procalcitonin assay, which is an enzyme-linked fluorescent immunoassay, performed in an automated Vidas instrument (BioMerieux Inc., Marcy l'Etoile, France).

### 2.8. Interleukins

The levels of IL-1*β*, IL-6, IL-8, IL-10, and TNF-*α* were determined with electrochemiluminescence method (Immulite 2000, Siemens, Erlangen, Germany) with respective commercial kits (Human IL-1*β* Elisa Kit, Human IL-6 Elisa Kit, Human IL-8 Elisa Kit, and Human IL-10 Elisa Kit, Sigma Aldrich, Interlab A.Ş., Istanbul, Turkey, and Plasma TNF-*α* immunosorbent assay test, Biosource, Invitrogen Corporation, Carlsbad, CA).

### 2.9. Statistics

The computer software used was SPSS 21.0 for Windows (SPSS Inc., Chicago, IL, USA). Results were expressed as mean and standard deviation or number and percent. Comparisons were carried out using Mann–Whitney *U* test for nonparametric data and *χ* ^2^-test or Fisher exact test for categorical variables. Group findings over different time were compared with repeated measures of analysis of variance. Group comparisons were carried out using Bonferroni test. Two-sided *p* value above 0.05 was considered statistically significant.

## 3. Results

The clinical data are listed in Tables 1 and 2. There were no statistically significant differences in any demographic, operative, or postoperative parameters between the groups. As depicted in Table 1, both of the study groups were found to be comparable for the presence of the risk factors [body mass index, insulin-dependent diabetes mellitus, systolic/diastolic blood pressure, hypercholesterolemia, smoking, recent MI, EF, and intraoperative ECC parameters (aortic cross-clamp time = AXT, CPB, and total surgery time)]. Both groups were comparable for mean AXT and mean duration of CPB, and *p* values were not significant (66.5 ± 18.27 versus 60.0 ± 13.93 and 116.27 ± 26.97 versus 113.5 ± 30.35, *p* = 0.26 and *p* = 0.79, resp.). All patients were extubated in the early postoperative period. No serious complications such as reoperation due to bleeding, thromboembolic event, MI, prolonged ICU or hospital stay, or mortality occurred during the study (Table 2).

**Figure 1:**
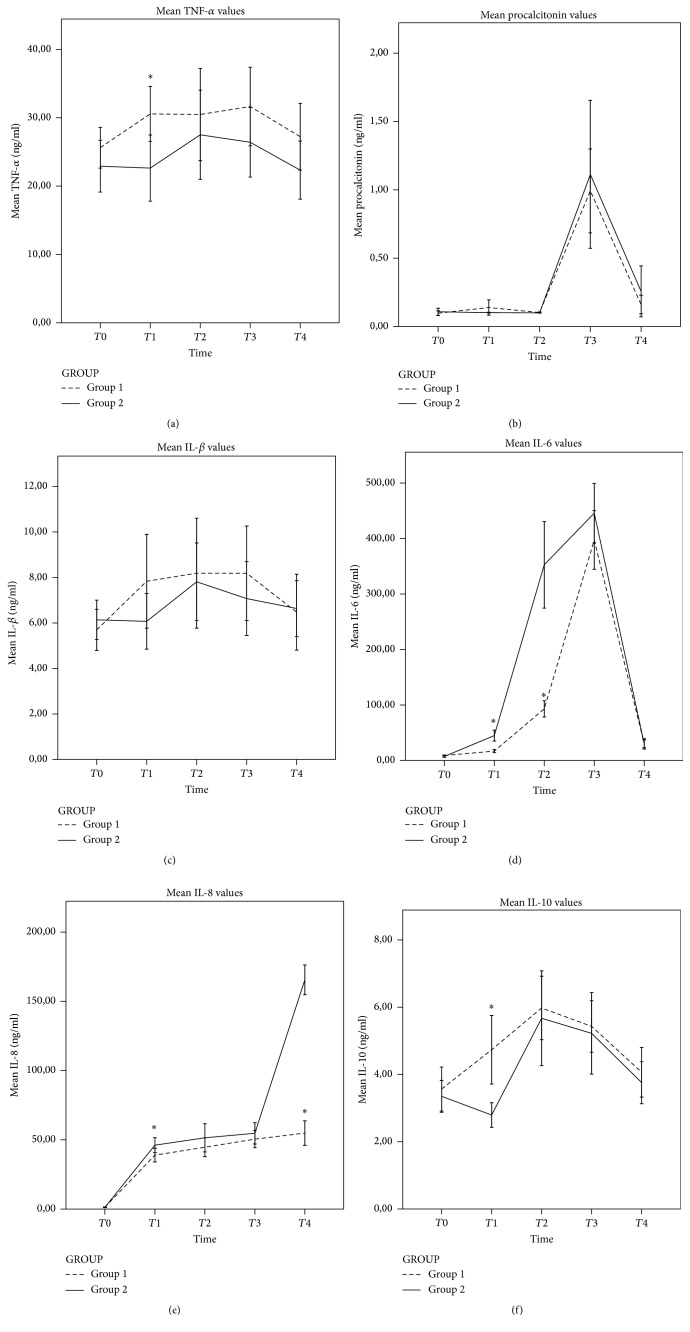
Graphs representing inflammation-related parameters expressed by Tumor Necrosing Factor-*α* (a), plasma procalcitonin (b), Interleukin-1*β* (c), Interleukin-6 (d), Interleukin-8 (e), and Interleukin-10 (f) of Group I patients (*n* = 18) and Group II patients (*n* = 14). Data presented show values at the moment of *T*0: preoperatively; *T*1: peroperatively just before cross-clamp removal; *T*2: peroperatively after termination of CPB; *T*3: postoperative 6th hour; and *T*4: postoperative 1st week. Significant differences (*p* < 0.05): *∗* versus Group II. Group I: phosphorylcholine-coated Extracorporeal Circulation Circuit, Group II: noncoated Extracorporeal Circulation Circuit, IL-1*β*: Interleukin-1*β*, IL-10: Interleukin-10, IL-6: Interleukin-6, IL-8: Interleukin-8, and TNF-*α*: Tumor Necrosing Factor-*α*.

TNF-*α* and IL-1*β* levels increased but this was not statistically significant compared to baseline levels in both groups (Group I *p* = 0.174 and *p* = 0.176, Group II *p* = 0.541 and *p* = 0.357, resp.) (Figures 1(a)–1(c)). The increase in TNF-*α* values (30.56 ± 8.11 versus 23.97 ± 7.8) was significant between the groups at *T*2 (*p* = 0.03) (Table 3). IL-6 and IL-8 values were significantly higher at all time courses compared to *T*0 in both groups (IL-6, Group I: 8.96 ± 1.94, 16.84 ± 5.63, 93.05 ± 29.63, 397.22 ± 106.41, and 29.88 ± 18.63 and Group II: 8.31 ± 1.92, 44.81 ± 17, 352.71 ± 135.11, 445.71 ± 92.71, 29.85 ± 12.3; IL8, Group I: 1.33 ± 0.52, 38.88 ± 9.8, 44.66 ± 13.62, 50.44 ± 12.55, and 54.85 ± 17.86 and Group II: 1.28 ± 0.42, 46.14 ± 9.25, 51.42 ± 17.60, 54.71 ± 13.35, and 165.5 ± 18.6, *p* < 0.001 for both groups) (Figures 1(d)-1(e)). The differences between the groups were significant at *T*2 for IL-6 and IL-8 (Group I versus Group II, 16.84 ± 5.63 versus 44.81 ± 17.0, *p* = 0.001, and 38.88 ± 9.8 versus 46.14 ± 9.25, *p* = 0.038, resp.) and at *T*3 for IL-6 (Group I versus Group II, 93.05 ± 29.63 versus 352.71 ± 135.11, *p* < 0.001) (Table 3). IL-10 value decreased in Group II (2.79 ± 0.63) but increased in Group I (4.73 ± 2.04) at *T*1 and this was statistically significant (*p* = 0.002) (Figure 1(f)). Procalcitonin values significantly increased at *T*3 (Group I: 0.09 ± 0.03 versus 0.99 ± 0.61, *p* = 0.014, and Group II: 0.1 ± 0.04 versus 1.11 ± 0.93, *p* = 0.001) (Figure 1(b)) but the difference between groups was not significant (*p* = 0.805) (Table 3).

**Figure 2:**
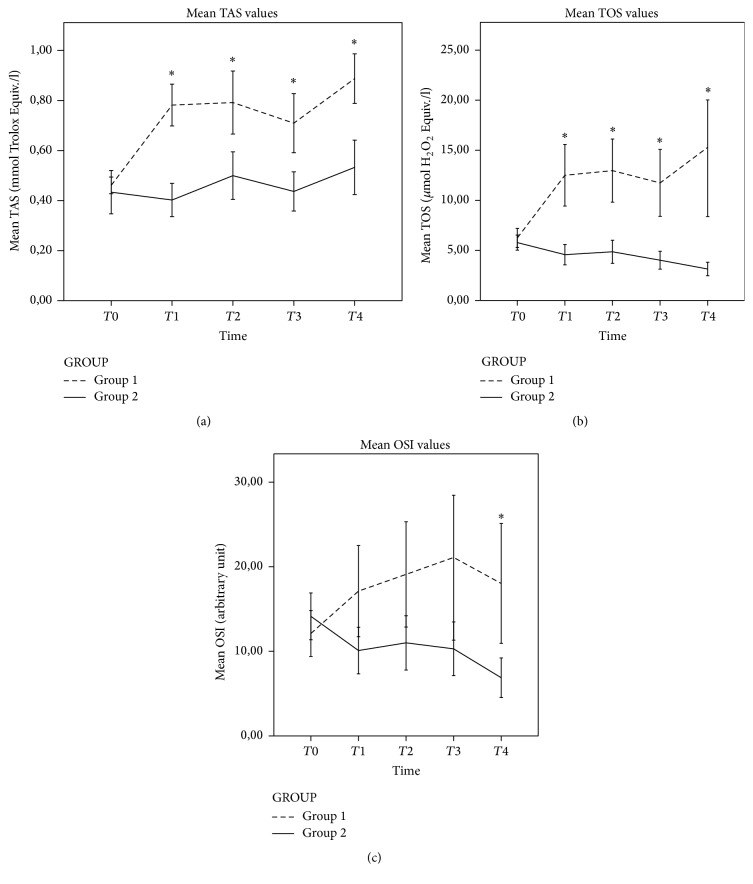
Graphs representing the oxidative stress expressed by TAS (a), TOS (b), and OSI (c) of Group I patients (*n* = 18) and Group II patients (*n* = 14). Data presented show values at the moment of *T*0: preoperatively; *T*1: peroperatively just before cross-clamp removal; *T*2: peroperatively after termination of CPB; *T*3: postoperative 6th hour; and *T*4: postoperative 1st week. Significant differences (*p* < 0.05): *∗* versus Group II. Group I: phosphorylcholine-coated Extracorporeal Circulation Circuit, Group II: noncoated Extracorporeal Circulation Circuit, TAS: Total Antioxidant Status, TOS: Total Oxidant Status, and OSI: Oxidative Stress Index.

Comparisons of the TAS, TOS, and OSI values between groups are shown in Table 2 and Figure 2. The TAS values (0.55 ± 0.12, 0.81 ± 0.2, 0.81 ± 0.3, 0.7 ± 0.23, and 0.88 ± 0.2, *p* < 0.001) significantly increased in Group I at different perioperative time points compared to preoperative values but the increase of Group II was similar to baseline (0.45 ± 0.17, 0.48 ± 0.13, 0.5 ± 0.16, 0.43 ± 0.13, and 0.53 ± 0.18, *p* = 0.188). Although TAS levels showed a negative correlation with hyperlipidemia and IL-6 and IL-8 levels at all time points [hyperlipidemia [*r* ^2^ (*p*)]: −0.389 (0.028 ), −0.402 (0.022), −0.409 (0.020), and −0.431 (0.014), IL-6 [*r* ^2^ (*p*)]: −0.506 (0.003), −0.471 (0.006), −0.392 (0.026), and −0.615 (<0.001), and IL-8 [*r* ^2^ (*p*)]: −0.665 (<0.001), −0.609 (<0.001), −0.561 (0.001), and −0.705 (<0.001), resp.], in contrast TAS levels showed a positive correlation with TNF-*α* and IL-10 at all-time points [TNF-*α* [*r* ^2^ (*p*)]: 0.405 (0.021), 0.457 (0.011), 0.405 (0.021), and 0.430 (0.014) and IL-10 [*r* ^2^ (*p*)]: 0.533 (0.002), 0.491 (0.004), 0.541 (0.001), and 0.434 (0.013), resp.] (Table 4).

TOS values significantly increased in Group I from the beginning of the operation through the end (*T*1: 6.24 ± 1.93, *T*2: 12.5 ± 6.18, *T*3: 12.96 ± 6.34, *T*4: 11.73 ± 6.71, and T5: 15.29 ± 11.71, *p* = 0.002) while they significantly decreased in Group II from the beginning to the end (*T*1: 5.77 ± 1.3, *T*2: 4.57 ± 1.76, *T*3: 4.85 ± 1.90, *T*4: 4.01 ± 1.55, and T5: 3.14 ± 1.16, *p* = 0.001). TOS values inversely correlated with IL-6 and IL-8 (IL-6 [*r* ^2^ (*p*)]: −0.640 (0.001), −0.587 (0.001) −0.514 (0.003), and −0.556 (0.001) and IL-8 [*r* ^2^ (*p*)]: −0.423 (0.016), −0.395 (0.025), −0.382 (0.031), and −0.507 (0.003), resp.] (Table 5).

## 4. Discussion

The aim of the present study was to investigate the effect of PCC ECC systems characterized with their biocompatibility leading to a diminished inflammatory response and oxidative/antioxidative status. The present study shows that the serum levels of IL-8 and IL-6 as proinflammatory cytokines were significantly increased in both groups following the initiation of CPB and remained elevated 1 week after the surgery. The other proinflammatory cytokines such as TNF-*α* and IL-1*β* showed an elevation from the baseline on group I but this was statistically not significant probably due to large standard deviations. Also PCT, an inflammatory indicator, showed a statistically significant sudden increase with CPB termination and returned to baseline after 1 week in both groups. The anti-inflammatory cytokine, IL-10, showed a similar pattern to that of proinflammatory cytokines, indicating that this is a natural defense mechanism upon exposure to harmful inflammatory situations such as CPB and cardiac surgery. The similar elevation and descent pattern of inflammatory and anti-inflammatory markers in both groups suggest that PCC may be unable to attenuate inflammatory responses to the CPB.

Total Antioxidant Status and TOS were analyzed to examine the oxidative stress and antioxidant stability induced during CPB, using a tip-to-tip PC coated system. Serum TOS and TAS levels were increased significantly during CPB and remained elevated 1 week after surgery in Group I. On the contrary, serum TOS and TAS were not significantly increased in the control group whereas a downward slope of TOS response was observed, indicating a higher oxidative stress together with an increased antioxidant effect in the PCC group (group 2). In other words, it has been demonstrated that ECC gives rise to a pronounced systemic inflammation with an induction in the oxidative stress compared to noncoated ECC systems.

The oxidative stress during CABG is very well-documented entity [8, 9]. In the last decade, using coated ECCs during CPB has gained widespread acceptance as a measure to reduce inflammatory response and thus to improve clinical outcomes [10]. There are only a few studies comparing the degree of oxidative stress in patients operated on with PCC versus noncoated ECC systems, and the results of these studies are debatable [5, 8, 9]. Sohn et al. examined the effects of the multiple biocompatible circuits on the inflammatory response and oxidative stress [5]. He concluded that PCC ECC systems may suppress inflammatory responses and may possess antioxidant effects, although only serum oxidized nitric oxide (NOx) and myeloperoxidase (MPO) levels were analyzed [5]. In the present study, we used TAS as a marker for Total Antioxidant Status in plasma. Unexpectedly, we did not document a suppressed TAS value or—in other words—a depletion of plasma antioxidants in both groups. While the TAS value was slightly increased (but not statistically significant) in noncoated ECC group, it was increased twofold in PCC coated ECC group (statistically significant). The nature of this increase is not known. The abundant plasma molecules with antioxidant activity are albumin and uric acid [3]. The main determinants of the TAS are these molecules. Albumin concentration in plasma, however, did not increase significantly during CPB (data not shown but decreased slightly) and could, therefore, not have contributed to the increase of TAS. The uric acid herein may contribute to increase of TAS [3]. Data is not shown in the study but, during the CPB, BUN increased significantly. Another possible cause of increased TAS could have been hemolysis of red blood cells during CPB and during or after sampling. Hemoglobin has been shown to interfere with the TAS measurements [3].

On the other hand, during CPB patient's body undergoes a serial sequence of events (hypothermia, hemodilution, arrest, ischemia, reperfusion, and extracorporeal circulation) which induce cell damage due to ATP breakdown and depletion of the naturally occurring defense mechanisms against free radical injury and endothelial function impairment, all contributing to the development of organ dysfunction [11]. These events are closely linked to inflammatory processes, including complement activation, cytokine release, and leukocyte activation along with induced adhesion molecule expression [11]. Numerous studies have described the nature of these ROS and the time course of their expression during CPB [4, 8]. This is closely connected with overproduction of ROS which is produced during the ischemia/reperfusion process and systemic inflammatory response, both of which are associated with cardiac surgery performed under CPB [11, 12]. Explanation for the primary mechanisms causing systemic inflammatory response (SIR) and oxidative stress during cardiac surgery includes the exposure of blood to synthetic material of ECC systems, the surgical trauma itself, and the sudden temperature changes of the body [8]. The close connection between ROS production, unphysiological circulation, and SIR during CPB is very well-known both with coated and noncoated ECC systems [13, 14]. Current studies have described the status of oxidative burden and the time course of their formation during CPB [15]. The nature of these oxidative events leads to depletion of plasma antioxidants, increased lipid peroxidation, and formation of other damaging metabolites [12, 16]. Antioxidant molecules prevent and/or inhibit these harmful reactions. Interestingly, in the present study, TAS and TOS results were not as expected, especially in the PCC ECC group as TOS value was increased significantly and, adversely, TAS value was not decreased in the PCC ECC group.

We found a negative correlation between TAS and hyperlipidemia, IL-6, and IL-8 (Table 4). Also there was a negative correlation between TOS and IL-6 and IL-8 (Table 5). The increase of proinflammatory mediators such as TNF-*α*, IL-6, and IL-8 may explain the depletion of plasma antioxidants [1, 3]. We also found that there was a significant positive correlation between TAS and TNF-*α* and IL-10. And even TNF-*α* is a proinflammatory mediator, and it is the corner stone molecule for the inflammatory or anti-inflammatory reactions [2]. Das et al. concluded that hyperlipidemia may induce to decrease in antioxidant status by decreasing the activity of the antioxidant enzymes such as SOD and glutathione peroxidase. The decreased SOD activity is probably the result of inactivation by lipid peroxyl radicals and their breakdown products [17].

Increased oxidative stress during CABG is also well-documented and explained [4, 5]. In addition, ECC, by increasing contact of blood with foreign substances, will induce systemic inflammatory responses associated with complement activation, cytokine release and cellular activation of neutrophils [10]. These are all sources of ROS production which will ultimately lead to depletion of plasma antioxidants. Coronary artery bypass surgery under CPB may possess an elevation in oxidant status and accordingly to this increase may raise antioxidant status [14].

In conclusion, oxidant and antioxidant status increase significantly during cardiac surgery performed under CPB independently of the ECC system used. We can conclude that patients undergoing cardiac surgery may be exposed to a potent oxidative stress and that their TAS is overproduced to engage TOS. Although PCC ECC systems are designed to improve hemacompatibility, our preliminary study' results showed that even coated systems are still incapable of overcoming subclinical inflammatory responses during cardiopulmonary bypass.

The limitation of our present study is that our data show higher standard deviations in serum cytokine levels and oxidative markers, which may be due to the fact that the immune responses differ widely between individuals.

## Figures and Tables

**Table 1:** Patient demographic and operative data.

	Group I (*n* = 18)	Group II (*n* = 14)	*p*
Age (years ± SD)	61 ± 11	61 ± 12	0.97
Gender (male, %)	53.8	46.2	0.56
Body mass index (kg/m^2^ ± SD)	22.72 ± 1.84	22.36 ± 1.19	0.71
Diabetes mellitus (%)	54.5	45.5	0.77
Systolic blood pressure (mmHg ± SD)	131 ± 17	138 ± 19	0.23
Diastolic blood pressure (mmHg ± SD)	66 ± 8	70 ± 8	0.22
Hyperlipidemia (%)	44.4	55.6	0.12
Smoking (%)	54.5	45.5	0.77
Recent myocardial infarction (%)	60	40	0.77
Ejection fraction (% ± SD)	49.94 ± 7.27	53.28 ± 7.8	0.22
Cross-clamp time (min ± SD)	66.5 ± 18.27	60.0 ± 13.93	0.26
Cardiopulmonary bypass time (min ± SD)	116.27 ± 26.97	113.5 ± 30.35	0.79
Total surgery time (min ± SD)	369.44 ± 57.67	373.92 ± 89.08	0.97

**Table 2:** Comparison of serum Total Oxidant Status, Total Antioxidant Status, and Oxidative Status Index in patients undergoing cardiac surgery with cardiopulmonary bypass circuit coated with phosphorylcholine Group I versus noncoated Group II at different time points.

		*T*0	*T*1	*T*2	*T*3	*T*4
TAS (mmoL of trolox/L, mean ± SD)	Group I (*n* = 18)	0.55 ± 0.12	0.81 ± 0.2	0.81 ± 0.3	0.7 ± 0.23	0.88 ± 0.2
	Group II (*n* = 14)	0.45 ± 0.17	0.48 ± 0.13	0.5 ± 0.16	0.43 ± 0.13	0.53 ± 0.18
*p* ^*∗*^		0.084	**0.003**	**0.001**	**0.002**	**0.001**

TOS (mean *μ*mol H_2_O_2_/L ± SD)	Group I (*n* = 18)	6.24 ± 1.93	12.5 ± 6.18	12.96 ± 6.34	11.73 ± 6.71	15.29 ± 11.71
	Group II (*n* = 14)	5.77 ± 1.3	4.57 ± 1.76	4.85 ± 1.90	4.01 ± 1.55	3.14 ± 1.16
*p* ^*∗*^		0.621	**0.001**	**0.001**	**0.003**	**0.001**

OSI (mean ± SD)	Group I (*n* = 18)	12.1 ± 5.45	17.11 ± 10.83	19.09 ± 12.51	21.09 ± 17.23	18.01 ± 14.23
	Group II (*n* = 14)	14.13 ± 4.79	10.09 ± 4.76	11 ± 5.55	10.29 ± 5.49	6.87 ± 4.05
*p* ^*∗*^		0.160	0.119	0.063	0.102	**0.048**

Continuous data were reported as mean ± standard deviation (SD) if normally distributed, as indicated by the shape of the distribution pattern in the Shapiro-Wilk test.

^*∗*^ *p* values for comparisons between Group I and Group II with Mann–Whitney *U* test. A *p* value of <0.05 is statistically significant. OSI: Oxidative Stress Index, TAS: Total Antioxidant Status, and TOS: Total Oxidant Status.

**Table 3:** Comparison of serum Interleukin-1*β*, Interleukin-10, Interleukin-6, Interleukin-8, procalcitonin, and Tumor Necrosing Factor-*α* in patients undergoing cardiac surgery with cardiopulmonary bypass circuit coated with phosphorylcholine Group I versus noncoated Group II at different time points.

		*T*0	*T*1	*T*2	*T*3	*T*4
IL-1*β* (mean ± SD)	Group I (*n* = 18)	5.7 ± 1.81	7.83 ± 4.14	8.18 ± 4.85	8.18 ± 4.18	6.47 ± 3.35
	Group II (*n* = 14)	6.13 ± 1.5	6.07 ± 2.11	7.80 ± 2.95	7.07 ± 2.8	6.63 ± 2.12
*p* ^*∗*^		0.402	0.468	0.530	0.518	0.361

IL-10 (mean ± SD)	Group I (*n* = 18)	3.56 ± 1.31	4.73 ± 2.04	5.97 ± 1.89	5.42 ± 1.54	4.06 ± 1.48
	Group II (*n* = 14)	3.35 ± 0.82	2.79 ± 0.63	5.67 ± 2.44	5.22 ± 2.09	3.75 ± 1.08
*p* ^*∗*^		0.954	**0.002**	0.479	0.181	0.351

IL-6 (mean ± SD)	Group I (*n* = 18)	8.96 ± 1.94	16.84 ± 5.63	93.05 ± 29.63	397.22 ± 106 41	29.88 ± 18.63
	Group II (*n* = 14)	8.31 ± 1.92	44.81 ± 17	352.71 ± 135.11	445.71 ± 92.71	29.85 ± 12.3
*p* ^*∗*^		0.472	**0.001**	**0.001**	0.161	0.506

IL-8 (mean ± SD)	Group I (*n* = 18)	1.33 ± 0.52	38.88 ± 9.8	44.66 ± 13.62	50.44 ± 12.55	54.85 ± 17.86
	Group II (*n* = 14)	1.28 ± 0.42	46.14 ± 9.25	51.42 ± 17.60	54.71 ± 13.35	165.5 ± 18.6
*p* ^*∗*^		0.411	**0.038**	0.177	0.065	**0.001**

Procalcitonin (mean ± SD)	Group I (*n* = 18)	0.09 ± 0.03	0.13 ± 0.11	0.1 ± 0.01	0.99 ± 0.61	0.16 ± 0.13
	Group II (*n* = 14)	0.1 ± 0.04	0.1 ± 0.01	0.1 ± 0.01	1.11 ± 0.93	0.25 ± 0.32
*p* ^*∗*^		0.451	0.652	0.378	0.805	0.389

TNF-*α* (mean ± SD)	Group I (*n* = 18)	25.62 ± 6.01	30.56 ± 8.11	30.47 ± 13.6	31.63 ± 11.55	27.22 ± 9.83
	Group II (*n* = 14)	23.17 ± 5.84	23.97 ± 7.8	26.92 ± 10.26	27.13 ± 8.15	22.33 ± 6.7
*p* ^*∗*^		0.282	**0.031**	0.594	0.396	0.164

Continuous data were reported as mean ± standard deviation (SD) if normally distributed, as indicated by the shape of the distribution pattern in the Shapiro-Wilk test.

^*∗*^ *p* values for comparisons between Group I and Group II with Mann–Whitney *U* test. A *p* value of <0.05 is statistically significant. IL-1*β*: Interleukin-1*β*, IL-10: Interleukin-10, IL-6: Interleukin-6, IL-8: Interleukin-8, and TNF-*α*: Tumor Necrosing Factor-*α*.

**Table 4:** TAS correlations.

*n* = 32	Hyperlipidemia	IL-10	IL-6	IL-8	TNF-*α*
*T*1					
Correlation coefficient	−0.389^*∗*^	0.533^*∗∗*^	−0.506^*∗∗*^	−0.665^*∗∗*^	0.405^*∗*^
*p*	0.028	0.002	0.003	0.000	0.021
*T*2					
Correlation coefficient	−0.402^*∗*^	0.491^*∗∗*^	−0.471^*∗∗*^	−0.609^*∗∗*^	0.457^*∗*^
*p*	0.022	0.004	0.006	0.000	0.011
*T*3					
Correlation coefficient	−0.409^*∗*^	0.541^*∗∗*^	−0.392^*∗*^	−0.561^*∗∗*^	0.405^*∗*^
*p*	0.020	0.001	0.026	0.001	0.021
*T*4					
Correlation coefficient	−0.431^*∗*^	0.434^*∗*^	−0.615^*∗∗*^	−0.705^*∗∗*^	0.430^*∗*^
*p*	0.014	0.013	0.000	0.000	0.014

*∗* represents  *p* < 0.05 and *∗∗* represents  *p* < 0.01 according to multivariate regression analysis.

**Table 5:** TOS correlations.

	IL-6	IL-8
*T*1		
Correlation coefficient	−0.640^*∗∗*^	−0.423^*∗*^
Sig. (2-tailed)	0.001	0.016
*T*2		
Correlation coefficient	−0.587^*∗∗*^	−0.395^*∗*^
Sig. (2-tailed)	0.001	0.025
*T*3		
Correlation coefficient	−0.514^*∗∗*^	−0.382^*∗*^
Sig. (2-tailed)	0.003	0.031
*T*4		
Correlation coefficient	−0.556^*∗∗*^	−0.507^*∗∗*^
Sig. (2-tailed)	0.001	0.003

*∗* represents  *p* < 0.05 and *∗∗* represents  *p* < 0.01 according to multivariate regression analysis.
